# Development and *in vitro* evaluation of κ-carrageenan based polymeric hybrid nanocomposite scaffolds for bone tissue engineering

**DOI:** 10.1039/d0ra07446b

**Published:** 2020-11-06

**Authors:** Muhammad Umar Aslam Khan, Mohsin Ali Raza, Hassan Mehboob, Mohammed Rafiq Abdul Kadir, Saiful Izwan Abd Razak, Saqlain A. Shah, Muhammad Zahir Iqbal, Rashid Amin

**Affiliations:** Department of Polymer Engineering and Technology, University of the Punjab 54590 Lahore Pakistan umar007khan@gmail.com; School of Biomedical Engineering and Health Sciences, Faculty of Engineering, Universiti Teknologi Malaysia 81300 Skudai Johor Malaysia; Department of Metallurgy and Materials Engineering, CEET, University of the Punjab Lahore Pakistan; Department of Engineering Management, College of Engineering, Prince Sultan University P. O. Box No. 66833, Rafha Street Riyadh 11586 Saudi Arabia; Center for Advanced Composite Materials, Universiti Teknologi Malaysia 81300 Skudai Johor Malaysia; Materials Science Lab, Department of Physics, Forman Christian College (University) Lahore Pakistan; Nanotechnology Research Laboratory, Faculty of Engineering Sciences, GIK Institute of Engineering Sciences and Technology Topi 23640 Khyber Pakhtunkhwa Pakistan; Department of Biology, College of Sciences, University of Hafr Al Batin 39524 Hafar Al-batin Saudi Arabia rashida@uhb.edu.sa

## Abstract

The excellent biocompatible and osteogenesis characteristics of porous scaffolds play a vital role in bone regeneration. In this study, we have synthesized polymeric hybrid nanocomposites *via* free-radical polymerization from carrageenan/acrylic-acid/graphene/hydroxyapatite. Porous hybrid nanocomposite scaffolds were fabricated through a freeze-drying method to mimic the structural and chemical composition of natural bone. Fourier-transform infrared spectroscopy (FTIR), scanning electron microscopy (SEM) and water contact-angle studies were carried-out for functional groups, surface morphology and hydrophilicity of the materials, followed by biodegradation and swelling analysis. The cell viability, cell culture and proliferation were evaluated against mouse pre-osteoblast (*MC3T3-E1*) cell lines using neutral red dye assay. The cell adherence and proliferation studies were determined by SEM. Physical characterization including optimum porosity and pore size (49.75% and 0.41 × 10^3^ μm^2^), mechanical properties (compression strength 8.87 MPa and elastic modulus 442.63 MPa), swelling (70.20% at 27 °C and 77.21% at 37 °C) and biodegradation (23.8%) were performed. The results indicated CG-*g*-AAc-3 with a high optical density and better cell viability. Hence, CG-*g*-AAc-3 was found to be more efficient for bone regeneration with potential applications in fractured bone regeneration.

## Abstract 2

The excellent biocompatible and osteogenesis characteristics of porous scaffolds play a vital role in bone regeneration.

## Graphical abstract

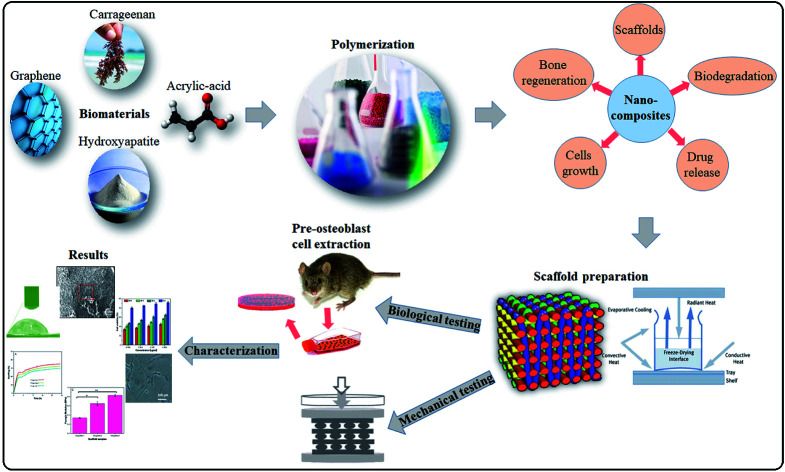

## 1. Introduction

Hybrid nanocomposite scaffolds provide considerable potential in bone tissue engineering for bone regeneration, cell adherence, migration, differentiation and proliferation *via* osteoproduction. The selection of biomaterials with brilliant physicochemical properties is very important to produce porous scaffolds.^1,2^ Another important parameter for bone reconstruction^3^ is the biodegradability of materials. Polysaccharide/hydroxyapatite composites signify the generic impact of the synthesis of biocompatible hybrid materials for bone tissue engineering.^4,5^ The incorporation of graphene oxide (GO) into reinforced polymer-ceramic hybrid composites has significantly improved the surface stability, mechanical properties and biocompatibility of the hybrid nanocomposite materials.^6^ Carrageenan (CG) is a water-soluble polysaccharide extracted from red algae which has several biomedical applications. The substantial amount of sulphonic groups in its structure make it a prominent biopolymer among all polysaccharides which imparts several biological properties due to the self-aggregation of helical structures.^7,8^ CG has been used primarily in the cosmetics, pharmaceutical, biomedical and food industries as an additive and stabilizer. To optimize and enhance the physiological characteristics of CG, mixing it with other polymers or with nano-reinforcing fillers is often needed for improved performance.^9^ In short, the structure of κ-carrageenan is identical to the polysaccharide and collagen of the body that can substitute the organic portion of the existing cytoskeleton of bone.^10,11^

Over the decade, the honeycomb structured graphene having the thickness of a carbon atom shows remarkable potential for biological applications which has gained considerable attention.^12^ Due to the biocompatibility of graphene, it has several biomedical applications including drug delivery, orthopedics, bioimaging and tissue engineering. Furthermore, hydroxyapatite (HAp) has its unique significance in developing hybrid scaffold systems for bone tissue engineering. HAp is a natural bone mineral with a hexagonal crystal structure which has less solubility than other calcium phosphates in biological conditions. These composite nano-materials may help to regulate cellular performances and bio-mineralization, and HAp can mimic the main inorganic minerals of the bone tissue.^13^ Considering the scientific value of graphene and hydroxyapatite in biomedical applications, their composite in optimum compositions is hypothesized to be giving promising results for better bone regeneration. The incorporation of graphene into hydroxyapatite based porous scaffolds may greatly enhance the biological as well as mechanical and physicochemical properties of the hybrid composite materials.^14^ Various efficient methods have already been utilized for the synthesis of nanocomposite biomaterials for bone substitutes. The free-radical polymerization is one of the effective techniques for synthesizing the polymeric nanocomposites due to its highly functional, controllable particle size and other physicochemical properties.^15^ Moreover, hybrid nanocomposite scaffolds can be fabricated using a freeze-drying method.^16^

**Fig. 1:**
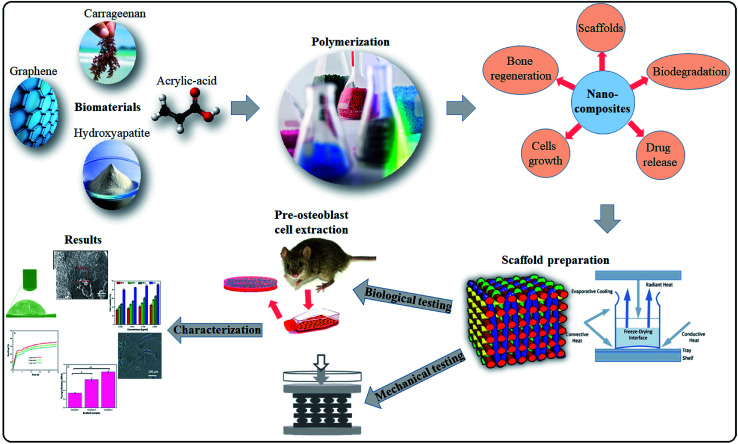
Presents in detail the synthesis of nanoparticles *via* free radical polymerization. Freeze drying technique was used to fabricate porous scaffolds and their comprehensive physicochemical and mechanical studies were to evaluate for bone tissue engineering.

In this study, the polymeric hybrid nanocomposites were synthesized to enhance the physiological properties and to promote cell growth based on surface morphology and microenvironment of the bone tissue. Carrageenan (CG) grafted acrylic acid (AAc), nano-hydroxyapatite (n-Hap) and graphene oxide (GO) doped hybrid nanocomposites were synthesized through free-radical polymerization technique. Porous hybrid nanocomposite scaffolds were fabricated *via* the freeze-drying method to investigate the swelling, porosity, mechanical properties, biodegradability, biocompatibility and antibacterial characteristics. The physicochemical and *in vitro* biological assay showed that these hybrid nanocomposite scaffolds possessed excellent biological characteristics to have potential in bone regeneration applications. The synthesis and characterizations of the porous hybrid nanocomposite scaffolds has been illustrated in Fig. 1.

## 2. Materials and methods

Carrageenan (C1013-100G), acrylic acid (AAc) (C_3_H_4_O), *N*,*N*′-methylene-bis-acrylamide (NN-MBA) (C_7_H_10_N_2_O_2_), potassium persulfate (K_2_S_2_O_8_), nano-hydroxyapatite (n-HAp) (<100 nm particle size, ≥95%), graphene oxide (GO) (763713-1G), Phosphate-Buffered Saline (PBS) solution and hydrochloric acid (HCl) were purchased from Sigma-Aldrich, Malaysia. All chemicals were used as received.

### 2.1. Synthesis of bioactive nanocomposite powder

Hybrid nanocomposites were synthesized by grafting of carrageenan and acrylic acid (CG-*g*-AAc) and doping n-HAp and GO through free-radical copolymerization technique. CG (2 g) was dissolved into deionized water and the solution was shifted into a three-necked round bottom flask. Then, acrylic acid (AAc) (0.50 mL) and *N*,*N*′-MBA (crosslinker) (0.05% of AAc by weight) were dissolved into the solution. Various amounts of GO (0.15, 0.20 and 0.25 mg) and n-HAp (2.5 g) were added slowly into the reaction media. The whole reactive solution was stirred for 2 h to prepare a homogeneous solution. Then K_2_S_2_O_8_ (0.05 g) as initiator was added and heated at 60 °C for 3 hours to initiate the reaction in the nitrogen environment. GO and n-HAp were doped into the polymeric network of arabinoxylan-grafted-acrylic acid (AX-*g*-AAc) giving rise to polymeric hybrid nanocomposites. Afterward, nitrogen flow was removed to cool the reaction media, then vacuum filtered. The residue was washed thoroughly with deionized water and overnight oven-dried at 55 °C to get fine powder of polymeric hybrid nanocomposites.

### 2.2. Fabrication of hybrid nanocomposite scaffolds

**Fig. 2:**
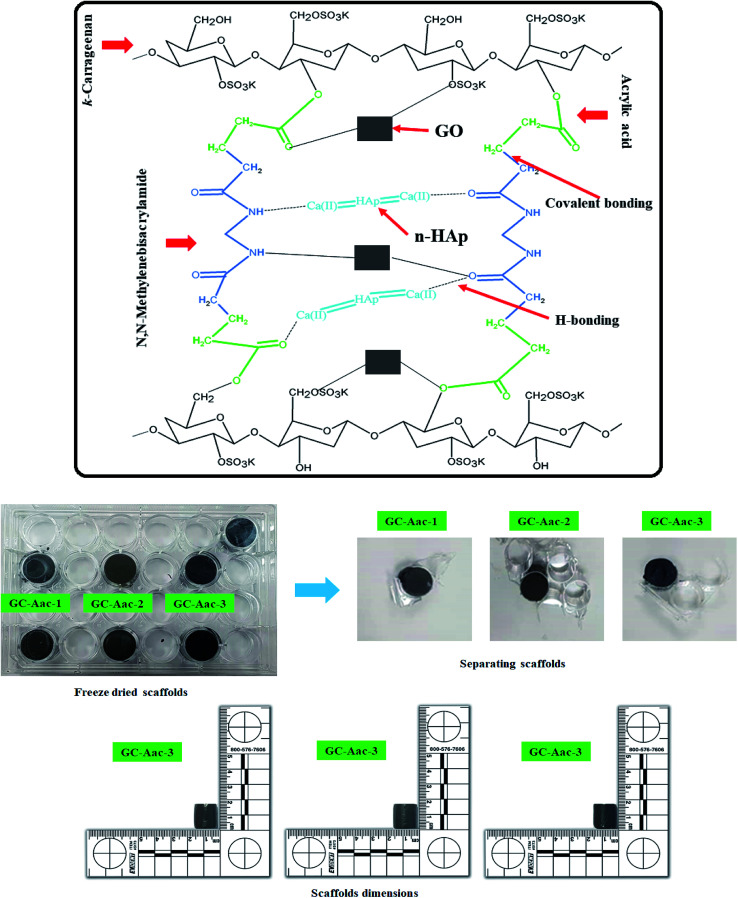
Presents the proposed chemical reaction and prepared hybrid nanocomposite scaffolds *via* the freeze-drying method for bone tissue engineering.

The hybrid nanocomposite scaffolds were fabricated using the freeze-drying method. Deionized water slurry was prepared from polymeric nanocomposite powder. The slurry was filled into 24 well plates (1.8 × 1.5 cm^2^), frozen at −80 °C for 24 h and then freeze-dried to get porous hybrid nanocomposite scaffolds. No crack or deformation was observed during and after the freeze-drying process. CG-*g*-AAc1, CG-*g*-AAc2 and CG-*g*-AAc3 names were assigned to the varying amounts of GO 0.15, 0.20 and 0.25 mg, respectively. The proposed chemical reaction and synthesis of hybrid nanocomposite scaffolds have been presented in Fig. 2.

## 3. Characterization

### 3.1. Fourier-transform infrared spectroscopy (FTIR)

Different functionalities of hybrid nanocomposite scaffolds were recorded using the FTIR (PerkinElmer Diamond 1000 spectrophotometer) in the range of 4000–400 cm^−1^.

### 3.2. X-ray diffractometer (XRD)

The crystalline behavior of the polymeric hybrid scaffolds was studied through an X-ray diffractometer (XRD). The XRD analysis was conducted by Bruker AXS D8 Advance XRD, the working voltage is 40 kV with current 30 mA. The Cu Kα radiation (1.540 Å) was used at 2*θ*° angle ranging from 20° to 80°.

### 3.3. Scanning electron microscope (SEM)

Surface morphologies of the hybrid nanocomposite scaffolds were studied using a scanning electron microscope (SEM-JSM 6940A).

### 3.4. Wetting

The hydrophilicity of the hybrid nanocomposite scaffolds was measured using contact-angle meter XCA-50. This measurement was done by dropping water droplets over the surface of the hybrid nanocomposite scaffolds and the photo was taken after 5 seconds. About 4 μL was the size of droplet and tests were carried out with three replicates.

### 3.5. Swelling and water holding capacity

The swelling analysis of dried hybrid nanocomposite scaffolds was carried out at different temperatures (27 and 37 °C) in aqueous and PBS media. Hybrid nanocomposite scaffolds (CG-*g*-AAc1, CG-*g*-AAc2 and CG-*g*-AAc3) were cut into square sizes. The initial weight of the dried scaffold (*W*_0_) was 40 mg. All hybrid nanocomposite scaffolds were dipped into a 250 mL beaker of PBS solution and deionized water at 37 °C and 7.4 pH. Afterward, these hybrid nanocomposite scaffolds were taken out from the corresponding media after regular intervals of time. These scaffolds were blot dried and final weight (*W*_f_) was measured. The percentage of swelling was calculated following.1
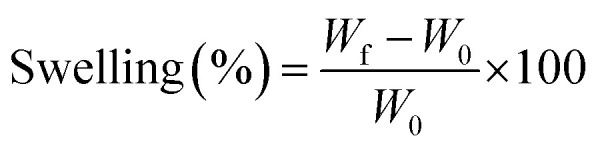


The water retention capacity of hybrid nanocomposite scaffolds was analyzed by filling water into scaffolds and centrifuged at 1000 rpm for 5 min. The water retention of hybrid nanocomposite scaffolds was measured using the following equation.2
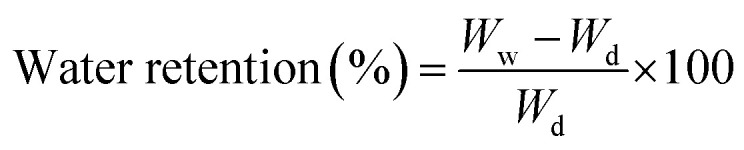
whereas *W*_d_ is the dry weight of scaffold and *W*_w_ is the wet weight of the scaffold.

The liquid displacement method was employed to determine the porosity by measuring diameter (*d*), height (*h*) and the dry weight (*W*_d_) of hybrid nanocomposite scaffolds.^17^ The scaffolds were then immersed in ethanol for 5 min and measured again the wet weight (*W*_w_). The scaffold porosity was measured using the following equation.3
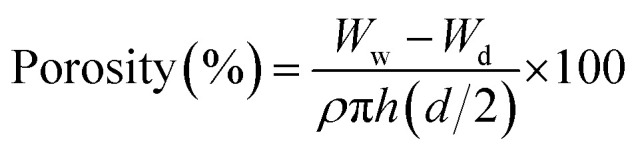
whereas *ρ* is the density of ethanol (0.789 g cm^−3^) and the value of π is 3.1416. *W*_w_ is the wet weight of scaffolds and *W*_d_ is the weight of dried scaffold.

### 3.6. Brunauer–Emmett–Teller (BET) analysis

The pore area of the hybrid nanocomposite scaffolds was examined by BET (Micromeritics Gemini II 2370).

### 3.7. Biodegradation

These hybrid nanocomposite scaffolds were kept in PBS solution (pH 7.4) and incubated at 37 °C for 30 days with their three replicates. Their degradation rate was determined until 30 days. Every day, these hybrid nanocomposite scaffolds were taken out from the PBS solution, rinsed with deionized water and kept into the oven for 1 hour at 37 °C. The weight loss of hybrid scaffolds was calculated using the following equation.4
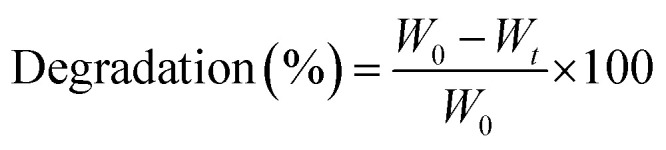
whereas *W*_*t*_ is weight at a specific time, *W*_0_ is the initial weight.

### 3.8. Mechanical testing

The hybrid nanocomposite scaffolds were cut into appropriate sizes (height = 1.6 cm and diameter = 1.5 cm) and the mechanical properties of hybrid nanocomposite scaffolds were analyzed by a universal testing machine (UTM, Testometrics, United Kingdom) with a loading rate of 5 mm min^−1^. The respective compressive modulus was determined as described previously.^18^

### 3.9. *In vitro* biological activities

#### 3.9.1. Cell culture and morphology

Mouse pre-osteoblast (*MC3T3-E1*) cell-lines were obtained from the American Type Culture Collection (ATCC-USA). Alpha-MEM (α-*MEM*) was purchased from Hyclone Laboratories Inc, and fetal bovine serum (*FBS*) and l-glutamine penicillin/streptomycin were purchased from ThermoFisher Scientific. *MC3T3-E1* cell-lines were maintained into α-*MEM* without ascorbic acid, 10% FBS, 1% (2 mM) l-glutamine and 1% penicillin/streptomycin. The density of *MC3T3-E1* cell lines was considered to be 5000 cells per cm^2^ in a 100 mm culture plate, whereas, gelatin (0.1% by conc.) was used as a coating agent. These cell lines were incubated along with scaffolds (CG-*g*-AAc1, CG-*g*-AAc2 and CG-*g*-AAc3) under standard *in vitro* conditions (37 °C, 5% CO_2_ and 90% humidity).

#### 3.9.2. Cell viability

Pre-osteoblast (*MC3T3-E1*) cell lines were cultured against different concentrations of scaffold extracts (0.50–2.00 mg mL^−1^) and 0.1% gelatin (+ive control). All the cells were incubated under standard *in vitro* conditions for 24, 48 and 72 h. These cultured cells were treated with neutral red assay as reported by Repetto *et al.*^19^ All these experiments were carried out in triplicate. These treated cells were incubated in a neutral red medium (40 μg mL^−1^) for 2 h. Then, these cells were washed with PBS solution after 2 h of incubation to remove the excessive neutral red stain. These cell lines were destained using the de-staining solution (50% distilled water, 49% absolute ethanol and 1% glacial acetic acid) at 37 °C for 10 min. The optical density was examined at 570 nm by an absorbance microplate reader (Bio-Tek, ELx-800, USA). The cell viability percentage was calculated by eqn (5).5
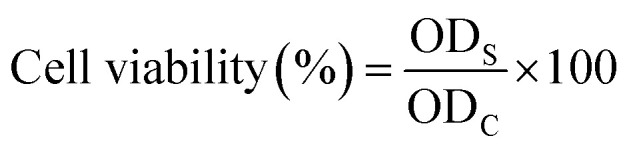
whereas OD_S_ is the optical density of sample concentration and OD_C_ is the optical density of the positive control.

#### 3.9.3. Cell culture and SEM morphological analysis

The cell culture and adherence along with surface morphology of all scaffold samples (CG-*g*-AAc-1, CG-*g*-AAc-2 and CG-*g*-AAc-3) were observed by SEM (JEOL-JSM-6480). The preosteoblast (*MC3T3-E1*) cell lines were cultured over scaffolds for different time intervals 24, 48 and 72 hours. These attached cells then washed using PBS solution to remove unattached cells and suspended particles. Later, these cells were fixed for 5 minutes at ambient using absolute ethanol. The well-dried scaffolds were gold-sputtered and SEM was operated at 1 kV, 7 × 10^−2^ bar operating pressure and 20 mA/2.0 min current deposition.

### 3.10. Statistical analysis

Experimental data was conducted in triplicate form and presented with mean standard errors (S.E). The statistical analysis was carried out using statistical tool software (IBM, SPSS Statistics 21). The means and standard errors of means (mean ± S.E) were calculated for every analysis, and S.E values were displayed as Y-error bars in figures. The error bars displayed standard deviations (*p* < 0.05 (5%); size of the sample *n* = 3).

## 4. Results and discussions

### 4.1. FTIR analysis

**Fig. 3:**
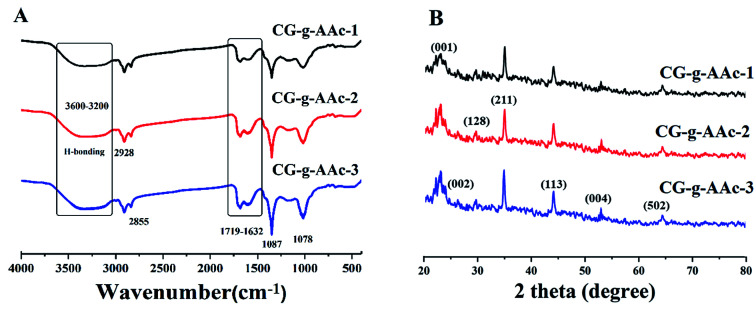
Presents the structural analysis of the polymeric hybrid nanocomposite scaffolds. (A) Shows the FTIR profile of different functional groups of all samples of scaffolds and (B) presents the XRD spectra of polymeric hybrid nanocomposite scaffolds to determine the crystalline behavior.

The FTIR spectral profile presented different functional groups of hybrid nanocomposite scaffolds of CG, AAc, GO and n-Hap, as shown in Fig. 3A. The broad bands 3600–3200 cm^−1^ and absorption peak at 1631 cm^−1^ are attributed to stretching and bending vibrations of hydrogen bonding and free and hydroxyl (–OH) groups.^20,21^ The absorption peaks at 1632 and 1714 cm^−1^ were assigned to C=C and C=O stretching vibrations are attributed to GO.^22^ Moreover, the broadband 3600–3200 cm^−1^ illustrates that CG, GO and AAc was connected by hydrogen bonding and peak at 2928 cm^−1^ attributed to saturated aliphatic C–H stretching vibrations.^4^ The bands at 1056 cm^−1^ is a characteristic cyclic peak due to polysaccharide. The spectral characteristic peak of 527 cm^−1^ is attributed to the calcium phosphate moiety of HAp.^23^ The band at 1078 and 969 cm^−1^ are attributed to the triply degenerated P–O stretching (the first one) and O–P–O bending (the latter two) of n-HAp. Moreover, the peak at 630 cm^−1^ also confirmed the occurrence of –OH.^4,24^ The presence of all these peaks clearly showed the successful synthesis of hybrid nanocomposite scaffolds.

### 4.2. XRD analysis

The XRD diffractograms and parameters of the GO/HAp/CG-*g*-AAc polymeric hybrid nanocomposites have been demonstrated in Fig. 3B. The characteristic peaks of HAp appeared at 2*θ* of 26.33, 29.76, 34.27, 44.14 53.04 and 64.52correspond to (002), (128), (211), (113), (004) and (502).^4,25^ However, the hydroxyapatite cell parameters are *a* = *b* = 9.4000 and *c* = 6.9300. These cell parameters are perfectly corresponding to the standard data (PDF-4-932) and the average crystallite size of HAp is 23.29 nm. Although, the diffraction peak at 2*θ* values of 23.16 corresponds to (001) confirm the presence of GO.^26^ The crystalline behavior of the CG-*g*-AAc decreases in the polymeric hybrid nanocomposites because there is no peak was for CG and AAc. The reducing crystal behavior of CG-*g*-AAc is due to the formation of hydrogen bonding, which is formed during free radical polymerization of polymeric components (CG, AAc and NN′, MBA) and engulfing of n-HAp and GO into extracellular like polymeric network.^4^

### 4.3. SEM and EDX

**Fig. 4:**
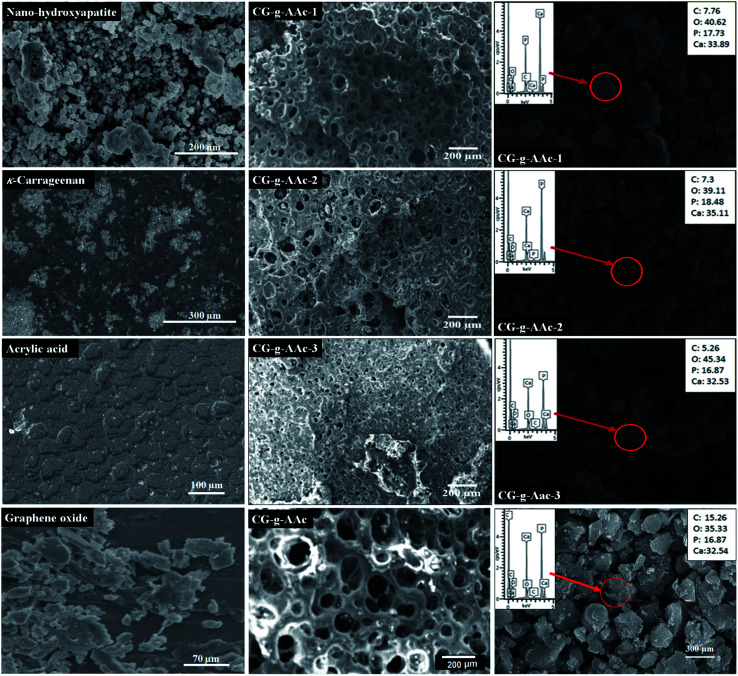
Surface morphologies of scaffolds, CG-*g*-AAc film and basic components (n-HAp, CG, AAc and GO) at different magnifications and EDX spectral analysis for the composition of elements in the scaffolds.

The scanning morphology of hybrid nanocomposite scaffolds was studied using SEM at 100 μm resolution and their micrographs are displayed in Fig. 4. All of these hybrid nanocomposite scaffolds demonstrated completely interconnected porosity with well-architectured foam-like morphology. This character is an important tissue ingrowth criterion due to cell adherence, growth and proliferation due to interconnected porosity and 50–250 μm best optimum pore size.^27,28^ All hybrid nanocomposite scaffolds in this analysis did not have cracks or other defects, indicating strong control over the manufacturing process. Thus, we successfully fabricated porous hybrid nanocomposite scaffolds. GO played a crucial role in the fabrication of porous hybrid nanocomposite scaffolds with rough morphology.^29^ The increasing amount of GO regulates the pore size and porosity uniformity. The pore size of CG-*g*-AAc1 was smaller than that of CG-*g*-AAc3 due to different amounts of GO that also increased the porosity of the scaffolds. The intensely interconnected porous composite structures are important for sustaining tissue fluid and the transportation of oxygen and nutrients are essentials for cell proliferation and migration in osteogenesis.^30^ The rough morphology and optimum pore size encourage cell adhesion, proliferation and migration for osteogenesis of osteoblast and osteoclast.^31^ The rough surface and larger pore size facilitate bone regeneration due to cell adherence and migration into the inter-connected hybrid nanocomposite scaffolds. Fig. 3 also presents the SEM morphologies of basic components *i.e.* nano-hydroxyapatite,^32^ κ-carrageenan,^33^ acrylic acid,^34^ graphene oxide^35^ and CG-*g*-AAc^36^ film.

### 4.4. Water contact-angle

**Fig. 5:**
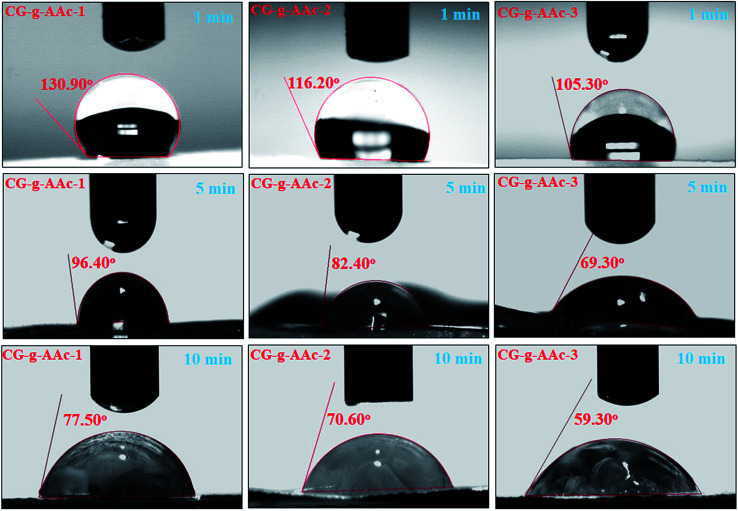
The measurement of the water contact angle to determine the hydrophilicity and hydrophobicity of scaffolds samples.

The comparison of water contact-angle among hybrid nanocomposite scaffolds is demonstrated in Fig. 5. The contact angles of polymeric hybrid nanocomposite scaffolds (CG-*g*-AAc-1, CG-*g*-AAc-2 a CG-*g*-AAc-3) were analyzed at a different time interval (1, 5 and 10 minutes) to determine the wetting behavior of polymeric hybrid scaffolds due to GO. The results showed that the hydrophilicity of hybrid nanocomposite scaffolds increased with the increase in GO content.^37^ The decreasing trend in contact-angle is a factor of an increasing amount of GO, though graphene was found to have few hydrophobic properties.^38,39^ Moreover, GO has unique hydrophilic characteristics due to hydrogen bonding at the interface.^40^ The droplet contact-angle of CG-*g*-AAc-1 was greater than that of CG-*g*-AAc-3 according to the amount of GO. Fig. 4 presents strong evidence of CG-*g*-AAc-3 to be hydrophobic than other scaffolds due to the maximum amount of GO. The increasing hydrophobicity trend was observed from CG-*g*-AAc-1 to CG-*g*-AAc-3. The increasingly hydrophilic character offers more hydrogen bonding which is a vital phenomenon for cell adhesion and cell proliferation.^41^ Therefore, it is presumed that the increasing amount of GO caused more hydrophilicity in the hybrid nanocomposite scaffold due to oxygen-based functional groups which facilitate the hydrogen bonding. An increasing certain amount of GO facilitates hydrogen bonding that tends to hydrophilic behavior of scaffolds. CG-*g*-AAc-3 was found to be more hydrophilic as compare to other scaffolds and the hydrophilic character increases as the time increased from 1 to 10 minutes. Hence, CG-*g*-AAc-3 was observed with maximum biological activity due to more interaction with the extracellular matrix than other samples.

### 4.5. Swelling, water retention, pore area and porosity

**Fig. 6:**
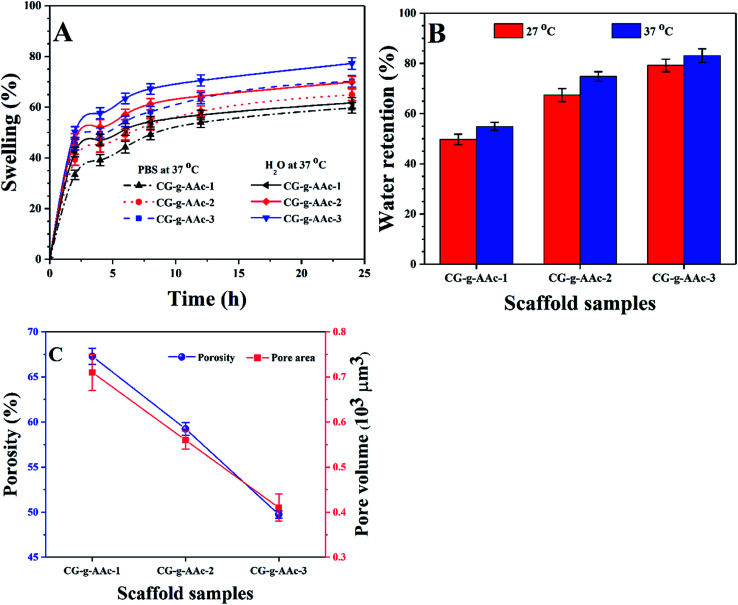
(A) Swelling analysis was conducted at 37 °C into aqueous and PBS media, (B) water retention capacity (at 27 and 37 °C) and (C) the porosity and pore volume of all samples of hybrid nanocomposite scaffolds and CG-*g*-AAc film. (*P < 0.05, **P < 0.01).

The swelling properties of all samples of scaffolds studied in aqueous and PBS media (Fig. 6A). Since swelling behavior of biomaterials is an essential function of scaffolds as it regulates cell nutrient, metabolism and gas exchange. The GO helped to regulate porosity, pore size and pore distribution that provided a much larger surface area of hybrid nanocomposite scaffolds which would help cell adherence, proliferation and differentiation. The increase in swelling offers more hydrogen bonding that enhances cell performance which is essential for bone regeneration.^42^ However, swelling under physiological conditions should be controllable as it is interlinked degradation of the bone scaffold.^43^ The correlation between aqueous and PBS media was found to be different because of the swelling behavior of hybrid nanocomposite scaffolds at 37 °C (Table 2). We have observed swelling, water retention, pore area and porosity trend among all hybrid nanocomposite scaffolds like CG-*g*-AAc-3 > CG-*g*-AAc-2 > CG-*g*-AAc-1 (Fig. 4A). CG-*g*-AAc-3 contains the largest amount of GO among all the scaffolds that had a higher degree of hydration. A hybrid nanocomposite scaffold with higher water retention capacity could enhance nutrients transfer and cell proliferation. From Fig. 5B, it was found that the CG-*g*-AAc-3 hybrid nanocomposite scaffold has the highest water retention capacity (Table 1) due to the most hydrophilic character and CG-*g*-AAc-1 has the least hydrophilic character due to the least amount of GO.^4,17^ Hybrid nanocomposite scaffolds having higher water retention capacity that helps to improve the transportation of nutrients and enhance cell migration, differentiation and proliferation.^18^ The CG-*g*-AAc-1, CG-*g*-AAc-2 and CG-*g*-AAc-3 scaffolds were found to be highly porous structure and their parameters have been described in Table 1 for all samples of scaffolds. The relationship between porosity and pore area is shown in Fig. 5C.

**Table 1:** The detailed values of swelling (27 and 37 °C) and water retention (27 and 37 °C) of all samples of the scaffold

Sample	Biodegradation (%)	Swelling (%)		Water retention (%)	
		27 °C	37 °C	27 °C	37 °C
CG-*g*-AAc-1	15.9 ± 1.1	59.70 ± 2.1	61.71 ± 2.1	49.70 ± 2.1	54.91 ± 1.6
CG-*g*-AAc-2	18.9 ± 1.2	64.87 ± 2.3	69.87 ± 2.3	67.40 ± 2.6	74.82 ± 1.9
CG-*g*-AAc-3	23.8 ± 1.1	70.20 ± 2.3	77.21 ± 2.3	79.20 ± 2.5	83.10 ± 2.7

### 4.6. Mechanical properties

**Fig. 7:**
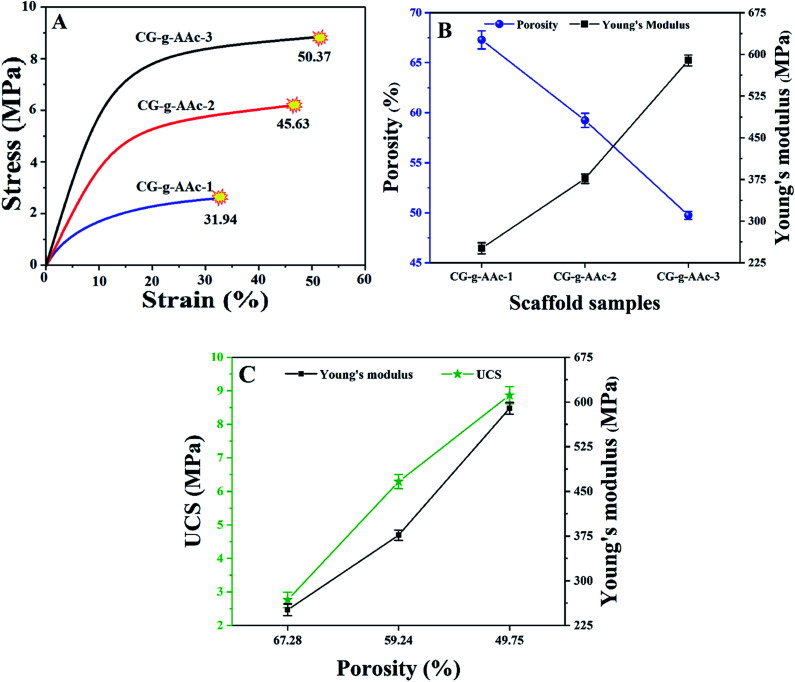
Mechanical properties of scaffolds obtained from compression tests; (A) stress–strain plot of compression tests, (B) relationship of porosity and Young's modulus and (C) porosity *vs.* ultimate compression strength and Young's modulus. (*P < 0.05, **P < 0.01).

The results of the mechanical properties of scaffolds were obtained from the compression tests as shown in Fig. 6. Stress–strain data was plotted for all types of hybrid nanocomposite scaffolds in Fig. 7A depicts that the scaffolds were completely fractured at the different stress and strain. These stress–strain plots were used to calculate Young's moduli of scaffolds. A Hook's law was used to calculate Young's modulus as shown in eqn (6).6
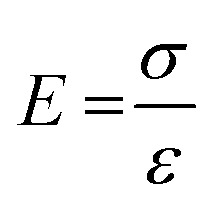
whereas *E* is the elastic modulus, *σ* is the stress and *ε* is the strain obtained from the linear region of stress–strain curves in Fig. 7A. Fig. 7B shows the relationship of porosity *versus* Young's moduli in CG-*g*-AAc-1, CG-*g*-AAc-2 and CG-*g*-AAc-3 scaffolds. The mechanical properties are shown in Fig. 7 and summarized in Table 2 of all scaffolds samples. The plot shows that the increase in scaffold porosity decreased the elastic Young's modulus and *vice versa*. The highest porosity was observed in the CG-*g*-AAc-1 scaffold with the lowest Young's modulus and the lowest porosity was observed in the CG-*g*-AAc-3 scaffold with the highest Young's modulus. The CG-*g*-AAc-2 scaffold shows the intermediate porosity with intermediate Young's modulus. Thus, an inversely proportional relationship was observed between the porosity and Young's modulus of the scaffolds. Moreover, the results also revealed that the increase in the amount of GO in hybrid nanocomposite scaffolds increased Young's modulus and strength. Fig. 7C shows the relationship between the porosity, Young's modulus and ultimate compression strength of scaffolds. From the results, this was revealed that the increase in porosity decreased Young's modulus and ultimate compression strengths. The CG-*g*-AAc-3 scaffold showed 370% and 194% Young's modulus and ultimate compression strength, respectively than that of the CG-*g*-AAc-1 scaffold (30 MPa and 4.23 MPa). However, the porosity of the CG-*g*-AAc-3 scaffold was 74% as compared to the CG-*g*-AAc-1 scaffold. Thus, the porosity and amount of GO showed an inverse relationship with the mechanical properties of the hybrid nanocomposite scaffolds.

**Table 2:** Describes the various mechanical and pore factors of all samples of scaffolds

Sample	Porosity (%)	Pore area (μm^2^)	UCS (MPa)	Strain (%)	Young's modulus (MPa)
CG-*g*-AAc-1	67.28 ± 4.2	0.71 × 10^3^	2.76 ± 1.1	31.9 ± 4.2	251.28 ± 3.1
CG-*g*-AAc-2	59.24 ± 5.7	0.56 × 10^3^	6.29 ± 1.2	45.8 ± 4.2	376.47 ± 5.4
CG-*g*-AAc-3	49.75 ± 4.1	0.41 × 10^3^	8.87 ± 1.1	50.71 ± 4.2	442.63 ± 6.3

### 4.7. Biodegradation

**Fig. 8:**
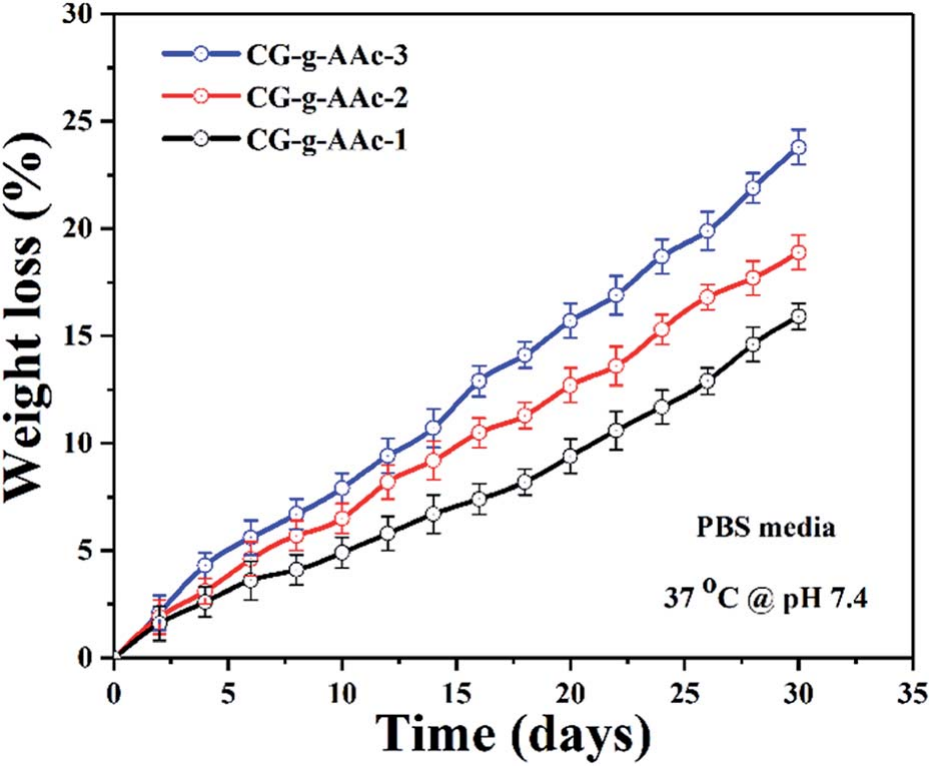
The degradation of all samples of hybrid nanocomposite scaffolds in PBS solution with pH 7.4 at 37 °C temperature.

The biodegradation characteristics of the hybrid nanocomposite scaffold were conducted in PBS solution at pH 7.4 and incubated at 37 °C. A below illustrates (Fig. 8) the results and biodegradation behavior of the hybrid nanocomposite scaffolds. Strong evidence of CG-*g*-AAc-3 was indicated high swelling property among all scaffolds and that may be due to more formation of hydrogen bonding between the media and scaffolds because of the increasing amount of GO.^44,45^ The biodegradation trend was observed in the order CG-*g*-AAc3 > CG-*g*-AAc2 > CG-*g*-AAc1 scaffolds. The increasing swelling cause more degradation and these both factors are correlated.^43^ The increasing amount of GO made the hybrid scaffolds more hydrophilic leading to maximum biodegradation of CG-*g*-AAc-3 under standard *in vitro* conditions due to more swelling.^46^ The biodegradation encourages osteogenesis due to cell adhesion, migration and proliferation due to surface properties and physicochemical characteristics.^47^ Nevertheless, the swelling capacity of the hybrid nanocomposite scaffolds was increased, with either the incorporation of GO. This is because GO should have increased hydrophilicity of CG-*g*-AAc-3 due to interaction with active hydrophilic groups Fig. 7.

### 4.8. *In vitro* activities

#### 4.8.1. Cell morphology

**Fig. 9:**
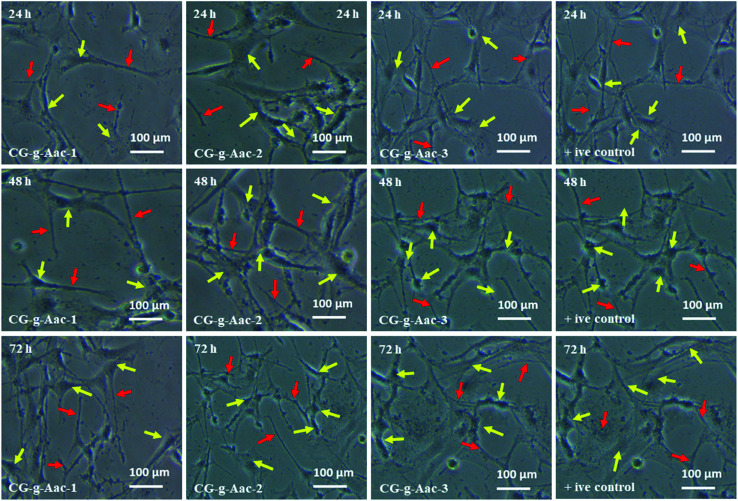
Cell morphology of *MC3T3-E1* against +ive control and all scaffold samples (CG-*g*-AAc1, CG-*g*-AAc2 and CG-*g*-AAc3) under standard *in vitro* conditions. The red arrows show thread-like morphology and the yellow arrows exhibits well-grown morphology of the cells.

*In vitro* biocompatibility of hybrid nanocomposite scaffolds was determined against *MC3T3-E1* cell lines. The results obtained from *MC3T3-E1* cell culture against hybrid nanocomposite scaffolds CG-*g*-AAc1, CG-*g*-AAc2 and CG-*g*-AAc-3 are presented in Fig. 9. The enhanced biocompatibility and cell differentiation on the substrate surfaces can be accomplished by activating different functionalities.^48^ As well, the increasing amount of GO also increased functionalities and surface area that enhanced the extent of *MC3T3-E1* adherence, differentiation and growth over the hybrid nanocomposite scaffold.^49^ The polymeric matric of hybrid nanocomposite scaffolds contains different chargeable functional groups (–COOH, –OSO_3_, –H, and –OH groups) and HAp has several active sites and revealed that the best cell adhesion, growth, and spreading. However little or no growth was observed over the negative control (DMSO).^50^ Although the surface morphology of all hybrid scaffolds is rough, interconnected, porous with different pore sizes than presented different cell performance, *MC3T3-E1* cells on the GC-*g*-AAc-3 found surfaces spread much more than GC-*g*-AAc1. Modulation of protein adsorption *via* integrin binding on negative modified surfaces can be used to regulate cell adhesion.^51^ Reports in the literature demonstrated that control of osteoblasts cells can be achieved *via* functionalization with ionizable groups that modulate the fibronectin adsorption and integrin-binding in the following trend –OH > –COOH > –NH_2_ > –CH_3_.^52^ After 24, 48 and 72 hours of culture at 37 °C, substantial changes in absorbance were noticed among all scaffold samples. Compared with control (0.1% gelatin-coating), CG-*g*-AAc-3 scaffolds showed higher cell viability after 72 h probably due to the physicochemical properties of hybrid nanocomposite scaffolds, which provided a supporting micro-environment to cells. The surface features of the scaffolds played a major role in cell adhesion which then promoted cell proliferation during the initial culture cycle. Furthermore, after 72 h there was substantial difference from CG-*g*-AAc-1 to CG-*g*-AAc-3 scaffolds. All cells were found to be cylindrical in shape, whereas red arrows present the thread like morphology and yellow arrows showed the well grown morphology of the MC3T3-E1. Hence, the thread like morphology of the cells were converted to well spread with the passage of the time. The possible reasoning is extracellular characteristics were slightly different but they had comparable porous structure.

#### 4.8.2. Cell viability and optical density

**Fig. 10:**
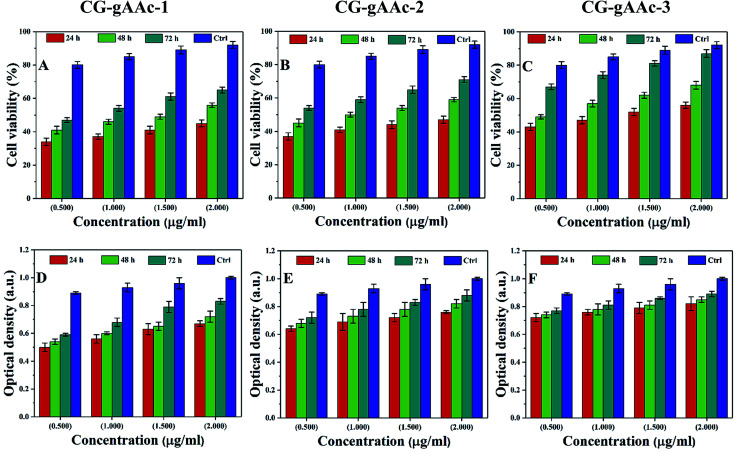
Cell viability and optical density of *MC3T3-E1* (A–F) against different concentrations (0.5, 1.0, 1.5 and 2.0 μg mL^−1^) of scaffolds and +ive control at different time intervals (24, 48 and 72 h) under standard *in vitro* conditions.

The cell proliferation of MC3T3-E1 is estimated *via* MTT assays as presented in Fig. 10. Since the optical density (OD) is based on the metabolic activities of MC3T3-E1 cells, it may be the total number of alive cells. So, the optical density is directly proportional to the metabolic activity of the alive cells.^53^ The cell viability assay (Fig. 10A–C) and optical density (Fig. 9D–F) of all hybrid nanocomposite scaffolds were studied against *MC3T3-E1* cell lines with different concentrations (0.5, 1.0, 1.5 and 2.0 μm mL^−1^) recorded after different time intervals (24, 48 and 72 h) under standard *in vitro* conditions. It was found (Fig. 10D–F) that cell proliferation was increased with increasing time and similar behavior was observed for optical density. The increasing value of optical density confirms the cytocompatible behavior of scaffolds against MC3T3-E1 cells.^53,54^ The scaffold (CG-*g*-AAc-1) has exhibited the highest OD values after 72 h among all samples, nearly to OD values of the control. Comparatively, CG-*g*-AAc-3 was found to biocompatible and will be a potential biomaterial for bone tissue engineering. Among all hybrid scaffolds, CG-*g*-AAc-3 presented maximum cell viability and proliferation. Therefore, the extract concentration 2 μg mL^−1^ was more apprehensive and showed better results. Hence, the reliability of our results supported our confidence that our hybrid nanocomposite scaffolds had cell viability, nontoxicity and proliferation towards pre-osteoblast cells.

#### 4.8.3. Cell culture

**Fig. 11:**
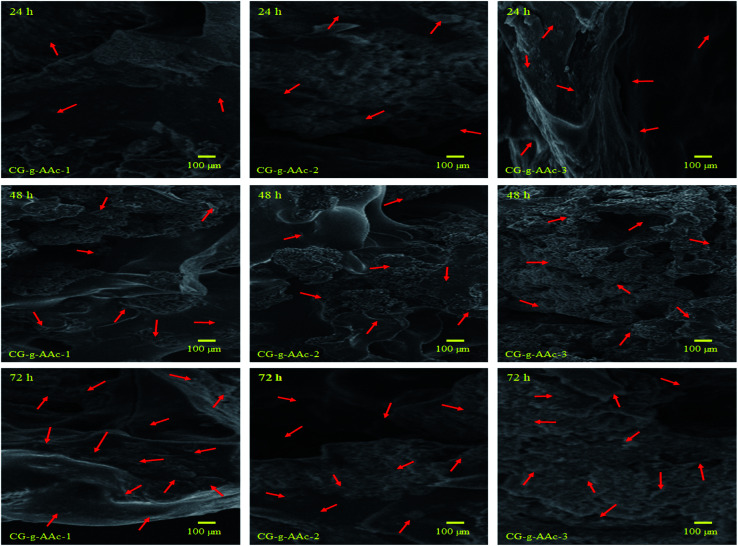
Presents the SEM images of the cell adherence against all samples of hybrid nanocomposite scaffolds at different time points (24, 48 and 72 h). The red arrow indicates cell adherence and SEM analysis helps to understand the behavior of pre-osteoblast cell lines against these scaffolds towards osteogenesis.

The preosteoblast (*MC3T3-E1*) cells were cultured over hybrid nanocomposite scaffolds to investigate the biocompatibility of the scaffolds (CG-*g*-AAc-1, CG-*g*-AAc-2 and CG-*g*-AAc-3). The cell adherence and proliferation characteristics of pre-osteoblast cells over the scaffolds were observed by SEM (Fig. 11). The growth and adherence of pre-osteoblast cells can be easily seen by red arrows over all scaffolds after 24, 48 and 72 hours after cell culture over the scaffolds. It was observed that more cells were adhered to after 72 hours and increasing the amount of GO. Though, CG-*g*-AAa-3 was observed with more cell proliferation and adhesion after an incubation time of 72 hours. Hence, cell culture and proliferation of MC3T3-E1 pre-osteoblast cell lines over CG-*g*-AAc-3 due to uniform porosity area, porosity and distributed interconnected pores (as mention in morphology analysis) that help cells migration and adherence over available active sites.^4,55^

## 5. Conclusion

Porous foam-like hybrid nanocomposite scaffolds were successfully fabricated from polymeric (CG, AAc, NN-MBA and GO) and ceramic (n-HAp) materials *via* copolymerization using the freeze-drying method. These hybrid nanocomposite scaffolds demonstrated different biomechanical properties including contact-angle, water retention swelling, biodegradation, pore size, Young's modulus, compressive strength and cell viability. Due to their different physicochemical behavior, these scaffolds showed different trends for cell culture. CG-*g*-AAc-3 is found a potential biomaterial due to considerable cell viability against pre-osteoblastic (*MC3T3-E1*) cells among all the scaffolds. These foam-like porous scaffolds demonstrated different physicochemical and *in vitro* biocompatibility properties due to different amounts of GO. Hence, it is concluded that the CG-*g*-AAc-3 hybrid nanocomposite scaffolds have the potential for bone tissue engineering.

## Conflicts of interest

There is no conflict among all authors.

## Supplementary Material
