# Correction: Development and *in vitro* evaluation of κ-carrageenan based polymeric hybrid nanocomposite scaffolds for bone tissue engineering

**DOI:** 10.1039/d1ra90119b

**Published:** 2021-05-24

**Authors:** Muhammad Umar Aslam Khan, Mohsin Ali Raza, Hassan Mehboob, Mohammed Rafiq Abdul Kadir, Saiful Izwan Abd Razak, Saqlain A. Shah, Muhammad Zahir Iqbal, Rashid Amin

**Affiliations:** Department of Polymer Engineering and Technology, University of the Punjab 54590 Lahore Pakistan umar007khan@gmail.com; School of Biomedical Engineering and Health Sciences, Faculty of Engineering, Universiti Teknologi Malaysia 81300 Skudai Johor Malaysia; Department of Metallurgy and Materials Engineering, CEET, University of the Punjab Lahore Pakistan; Department of Engineering Management, College of Engineering, Prince Sultan University P. O. Box No. 66833, Rafha Street Riyadh 11586 Saudi Arabia; Center for Advanced Composite Materials, Universiti Teknologi Malaysia 81300 Skudai Johor Malaysia; Materials Science Lab, Department of Physics, Forman Christian College (University) Lahore Pakistan; Nanotechnology Research Laboratory, Faculty of Engineering Sciences, GIK Institute of Engineering Sciences and Technology Topi 23640 Khyber Pakhtunkhwa Pakistan; Department of Biology, College of Sciences, University of Hafr Al Batin 39524 Hafar Al-batin Saudi Arabia rashida@uhb.edu.sa

## Abstract

Correction for ‘Development and *in vitro* evaluation of κ-carrageenan based polymeric hybrid nanocomposite scaffolds for bone tissue engineering’ by Muhammad Umar Aslam Khan *et al.*, *RSC Adv.*, 2020, **10**, 40529–40542. DOI: 10.1039/D0RA07446B.

The authors regret errors in Fig. 9 in the original article. The corrected Fig. 9 is shown below where all three +ive control panels and the 72 h CG-*g*-Aac-2 panel have been replaced.

**Fig. 9:**
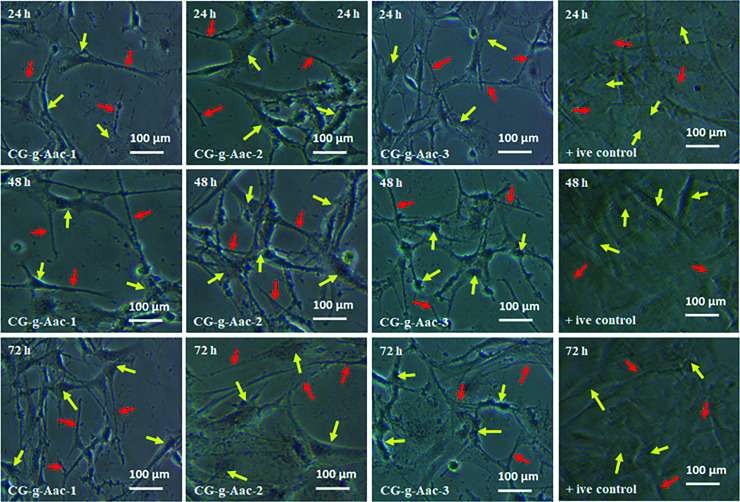
Cell morphology of *MC3T3-E1* against +ive control and all scaffold samples (CG-*g*-AAc1, CG-*g*-AAc2 and CG-*g*-AAc3) under standard *in vitro* conditions. The red arrows show thread-like morphology and the yellow arrows exhibits well-grown morphology of the cells.

The Royal Society of Chemistry apologises for these errors and any consequent inconvenience to authors and readers.

## Supplementary Material

